# The pathogenesis, therapeutic targets and drugs of polycystic ovary syndrome

**DOI:** 10.3389/fendo.2025.1722649

**Published:** 2026-01-26

**Authors:** Yawen Chen, Xiaoxiang Sun, Xuan Xia, Kaiqi Chen, Fang Zeng

**Affiliations:** Department of Pharmacy, Union Hospital, Tongji Medical College, Huazhong University of Science and Technology, Wuhan, China

**Keywords:** epigenetic modifications, gut microbiota, metabolic dysfunction, polycystic ovary syndrome, therapeutic targets and drugs

## Abstract

Polycystic ovary syndrome (PCOS) is a prevalent endocrine and metabolic disorder characterized by a high incidence rate and multiple complications, posing significant threats to women’s health and quality of life. The etiology of PCOS involves a complex interplay of genetic, metabolic, hormonal, immunological and environmental factors, though its precise mechanisms remain incompletely understood. This review explores the roles of oxidative stress, autophagy, ferroptosis, epigenetic modifications, post-translational modifications, chronic low-grade inflammation, and gut microbiota in the pathogenesis of PCOS. Current therapeutic strategies often combine lifestyle modifications with pharmacological interventions to address the multifaceted symptoms of PCOS. Drawing on the latest research, this review highlights advanced glycation end products (AGEs), sex hormone-binding globulin (SHBG), and microRNAs (miRNAs) as promising targets for PCOS prevention and treatment. Future research should focus on developing targeted drugs for these molecular pathways, offering new avenues for managing PCOS. This review will provide a scientific foundation for advancing PCOS treatment strategies.

## Graphical Abstract

**Figure:**
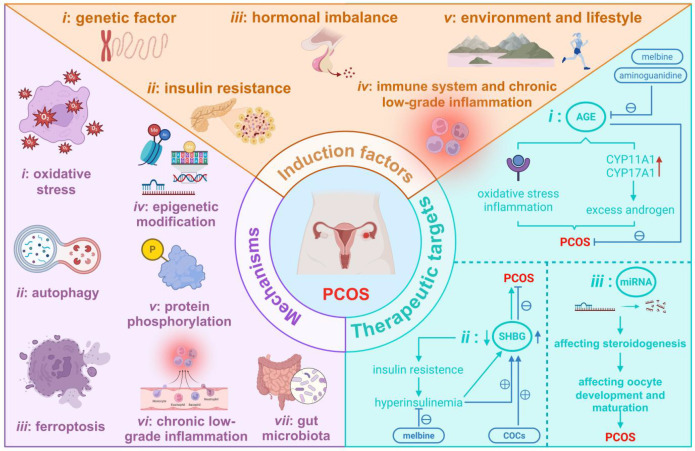

## 1 Introduction

**Figure 1:**
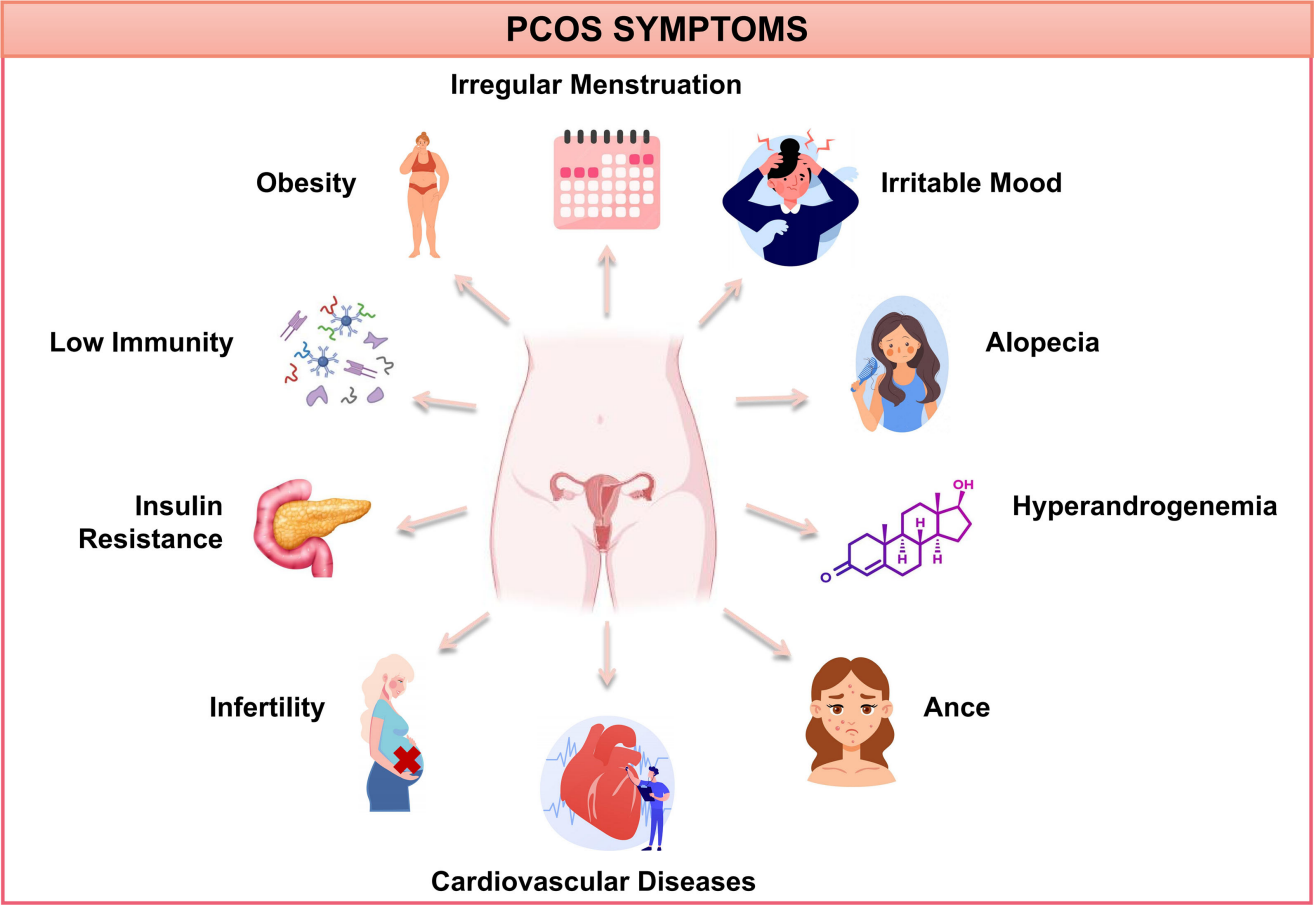
PCOS symptoms. PCOS, polycystic ovary syndrome.

Polycystic ovary syndrome (PCOS) is one of the most common endocrine and metabolic disorders in women of reproductive age, affecting millions worldwide. Epidemiological studies report a prevalence ranging from 4% to 21%, with approximately 11–13% of reproductive-aged women affected (1). The hallmark features of PCOS include menstrual irregularities, hyperandrogenism, and polycystic ovarian morphology. Additionally, PCOS is frequently associated with insulin resistance, impaired glucose tolerance, type 2 diabetes, obesity, cardiovascular diseases, dyslipidemia, *etc.* (**Figure 1**). These comorbidities not only elevate health risks but also contribute to infertility and diminished quality of life. For instance, insulin resistance affects 50–80% of women with PCOS, while impaired glucose tolerance and type 2 diabetes occur in 35% and 10% of cases, respectively. Obesity is prevalent in approximately 40% of PCOS patients, with 70% of obese individuals exhibiting at least one abnormal lipid parameter (2). Given its significant health burden, elucidating the mechanisms underlying PCOS is crucial for developing effective prevention and treatment strategies.

## 2 Factors affecting the occurrence and development of PCOS

The pathogenesis of PCOS is multifactorial, involving genetic predisposition, metabolic dysregulation, endocrine abnormalities, immune imbalance, and environmental influences, all of which interact to contribute to the disease phenotype.

### 2.1 Genetic factors

PCOS patients often have a family history and show a certain degree of familial clustering, suggesting a strong genetic component. Twin studies on small cohorts of monozygotic and dizygotic twins indicate that PCOS is not a monogenic disorder but rather a polygenic condition with X-linked inheritance patterns (3). Familial analyses have established that hyperandrogenemia in PCOS patients has a genetic foundation, further supporting the observed familial clustering of this condition (4).

Advances in genomic research have substantially enhanced our understanding of PCOS genetics. Genome-wide association studies (GWAS) have identified 29 susceptibility loci, which link PCOS to metabolic disorders, particularly those associated with elevated body mass index (BMI). Rare variants in Anti-Müllerian hormone (AMH) 2 and DENND1A have also been implicated in PCOS pathogenesis (5, 6). Furthermore, genetic polymorphisms affecting hormone regulation, insulin signaling, and inflammatory pathways may increase disease susceptibility (7). Collectively, these findings underscore the polygenic and multifactorial nature of PCOS, with genetic predisposition interacting with metabolic and endocrine factors to drive disease manifestation.

### 2.2 Insulin resistance

Insulin resistance is a central metabolic abnormality in PCOS, affecting 50–70% of patients irrespective of body weight. Hyperinsulinemia resulting from insulin resistance stimulates excessive androgen production by the ovaries and adrenal glands, exacerbating hyperandrogenemia and ovulatory dysfunction (8). Beyond its reproductive consequences, insulin resistance also contributes to dysregulated glucose and lipid metabolism, chronic inflammation. These derangements significantly elevate the risk of metabolic syndrome, type 2 diabetes, cardiovascular disease, and non-alcoholic fatty liver disease in affected individuals (9). The bidirectional relationship between insulin resistance and hyperandrogenemia creates a vicious cycle, wherein androgen impair insulin sensitivity, further aggravating metabolic dysfunction through affect fat distribution and muscle metabolism (10). Lifestyle interventions (*e.g*., diet and exercise) and pharmacological treatments (*e.g*., metformin, GLP-1 receptor agonists) can ameliorate insulin resistance and improve metabolic outcomes in PCOS (1, 11). These approaches underscore the central role of insulin resistance management in comprehensive PCOS care.

### 2.3 Hormonal imbalance

**Figure 2:**
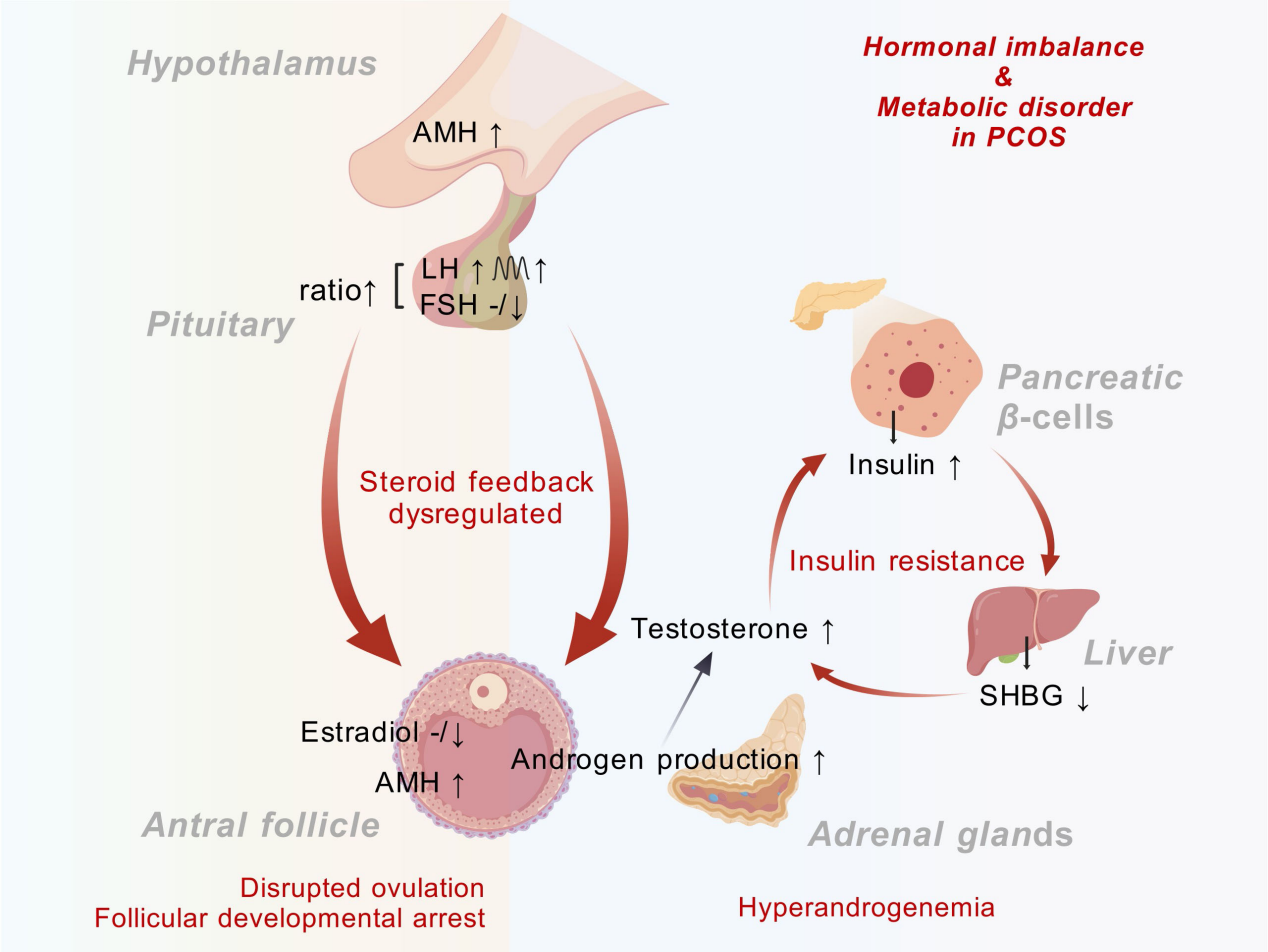
Metabolic-endocrine crosstalk in PCOS. PCOS, Polycystic ovary syndrome; AMH, Anti-Müllerian hormone; LH, luteinizing hormone; FSH, follicle-stimulating hormone; SHBG, sex hormone-binding globulin.

Hyperandrogenemia is a defining feature of PCOS, characterized by elevated serum testosterone, androstenedione, and dehydroepiandrosterone (DHEA) levels, leading to clinical manifestations such as hirsutism, acne, and alopecia. The mechanism mainly involves increased androgen synthesis in the ovaries and adrenal glands, along with a decrease in sex hormone-binding globulin (SHBG) levels, further increasing free androgen bioavailability (12). PCOS patients often exhibit elevated luteinizing hormone (LH) levels with normal or low follicle-stimulating hormone (FSH), resulting in an increased LH/FSH ratio and disrupted ovulation. PCOS patients may also have dysfunction of the hypothalamic-pituitary-ovarian (HPO) axis, increasing the LH pulse frequency and amplitude and reducing the FSH secretion (13). AMH levels are also elevated in PCOS due to the accumulation of small antral follicles, contributing to follicular developmental arrest (14). This excess AMH production further disrupts the delicate hormonal equilibrium in PCOS. The interplay between hyperandrogenemia and metabolic disturbances, such as insulin resistance and obesity, exacerbates the PCOS phenotype. Hyperandrogenemia promotes visceral adiposity and alters muscle metabolism, thereby exacerbating insulin resistance. Conversely, insulin resistance stimulates ovarian and adrenal androgen production through multiple mechanisms, creating a self-perpetuating pathogenic cycle (10). This vicious cycle underscores the intricate interplay between endocrine and metabolic dysfunction in PCOS (**Figure 2**).

### 2.4 Immune system and chronic low-grade inflammation

Chronic low-grade inflammation is a central feature of immune dysfunction in PCOS. Patients exhibit a persistent, mild, systemic inflammatory state even in the absence of infection. In women with PCOS, circulating levels of inflammatory markers such as C-reactive protein (CRP), interleukin-6 (IL-6), and tumor necrosis factor-α (TNF-α) are significantly elevated (15). Adipose tissue, particularly visceral fat, secretes large amounts of inflammatory factors (16). Hyperinsulinemia resulting from insulin resistance can itself stimulate inflammatory pathways. Dysbiosis of the gut microbiota may increase intestinal permeability, leading to the translocation of endotoxins (e.g., lipopolysaccharide, LPS) into the bloodstream and triggering immune responses (17). Secondly, immune cell dysfunction can also contribute to the development of PCOS. In adipose and ovarian tissues, macrophages are abnormally activated and polarized toward a pro-inflammatory (M1) phenotype, secreting IL-6, TNF-α, and other factors that exacerbate local and systemic inflammation and insulin resistance, potentially interfering with follicular development (18). The number or activity of neutrophils and mast cells is increased, further contributing to the inflammatory response. Studies have shown that PCOS patients exhibit an elevated proportion of Th17 cells and a reduced proportion of regulatory T cells (Treg). Th17 cells secrete pro-inflammatory cytokines such as IL-17, while Treg cells are responsible for maintaining immune tolerance and suppressing excessive inflammation (19). This imbalance (an increased Th17/Treg ratio) is a typical feature of autoimmune and chronic inflammatory diseases, disrupting immune homeostasis and promoting inflammation in ovarian and metabolic tissues. Immune factors do not act in isolation but form a vicious cycle with other characteristics of PCOS, such as insulin resistance and hyperandrogenism (20). They not only contribute to the pathogenesis of PCOS but also drive the progression of its clinical manifestations and the development of long-term complications.

### 2.5 Environment and lifestyle

Environmental exposures and lifestyle choices significantly influence PCOS risk. For example, active or passive smoking induces oxidative stress and systemic inflammation, impairing ovarian function and insulin sensitivity, thereby exacerbating metabolic and reproductive dysfunction in PCOS (21). Sleep disturbances, such as sleep apnea, contributes to sympathetic overactivation, oxidative stress, and heightened insulin resistance, further perpetuating chronic inflammation (22). Psychological stress may increase the stress hormone (such as cortisol) levels through hypothalamic-pituitary-adrenal (HPA) axis, leading to insulin resistance and increasing androgen secretion, further worsening PCOS symptoms (23). Dietary habits also critically influence PCOS pathogenesis. Excessive sugar intake exacerbates insulin resistance and low-grade inflammation, while a high-fat diet amplifies oxidative stress and adipose tissue inflammation (24). Conversely, vitamin D deficiency may aggravate insulin resistance and inflammatory responses in PCOS, whereas adequate vitamin D levels support metabolic and reproductive health by modulating insulin signaling and immune function (25). Given these connections, comprehensive lifestyle interventions, including dietary modifications, physical activity, and stress management, can improve metabolic and reproductive outcomes in PCOS (11).

## 3 Mechanism of PCOS occurrence

At present, the mechanism of PCOS occurrence is still unclear. Emerging research highlights the roles of oxidative stress, autophagy, ferroptosis, epigenetic modifications, post-translational protein modifications, chronic low-grade inflammation, and gut microbiota dysbiosis in PCOS development.

### 3.1 Oxidative stress

Oxidative stress occurs when the physiological equilibrium between oxidation and antioxidation is disrupted, resulting in a shift toward excessive oxidation. This state promotes neutrophil-mediated inflammatory infiltration, enhances protease secretion, and elevates the generation of oxidative intermediates. As a harmful consequence of free radical accumulation, oxidative stress is frequently observed in patients with PCOS, characterized by elevated reactive oxygen species (ROS) production and diminished antioxidant capacity. Concurrently, insulin resistance, chronic inflammation, and obesity contribute to ROS overproduction, while the activity of antioxidant enzymes (such as superoxide dismutase and glutathione peroxidase) are significantly reduced (26).

In PCOS, excessive ROS inflict damage on oocytes and granulosa cells, disrupt the follicular microenvironment, and impair follicular development and ovulation (27). Furthermore, ROS upregulate androgen-synthesizing enzymes (*e.g*., CYP17) in ovarian theca cells, exacerbating hyperandrogenemia (28). ROS also interfere with insulin signaling by inhibiting tyrosine phosphorylation of IRS, thereby worsening insulin resistance in PCOS patients (29). Elevated ROS levels in PCOS are associated with endothelial dysfunction and accelerated atherosclerosis, increasing cardiovascular risk (30, 31). Additionally, hyperandrogenism in PCOS induces mitochondrial oxidative stress in white adipose tissue, further aggravating insulin resistance and obesity (32). Therapeutic administration of antioxidants (*e.g*., vitamin C, vitamin E, N-acetylcysteine) may mitigate oxidative damage, improve insulin sensitivity, and restore ovarian function by neutralizing ROS (33).

### 3.2 Autophagy

Autophagy is a fundamental cellular process responsible for degrading and recycling damaged organelles and proteins, playing a critical role in maintaining cellular homeostasis. Emerging evidence suggests that autophagy may significantly contribute to the pathogenesis of PCOS (34). This process influences key pathophysiological mechanisms in PCOS, including follicular development, hormonal regulation, and metabolic balance. During early follicular development, the follicular pool undergoes dynamic changes, marked by an increase in both primary and antral follicles. Autophagy regulates granulosa cell apoptosis in early-stage follicles, contributing to follicular atresia. In cumulus cells, mitophagy is upregulated in response to follicular fluid, and its impairment can lead to oocyte dysfunction and morphological abnormalities. Furthermore, autophagy inhibits the transition of antral follicles to the pre-ovulatory stage, ultimately disrupting ovulation. Elevated levels of insulin, LH, and testosterone exacerbate PCOS progression by increasing intracellular cyclic adenosine monophosphate (cAMP) levels (35).

Beyond follicular dynamics, autophagy is essential for maintaining normal oocyte and granulosa cell function, as well as modulating insulin signaling (36). Dysregulated autophagy contributes to aberrant follicular development, anovulation (37), and insulin resistance (38) in PCOS patients. Additionally, impaired autophagy may disrupt steroidogenic enzyme activity in the ovaries and adrenal glands, leading to excessive androgen production. Autophagy also interacts closely with inflammatory pathways. By suppressing NOD-, LRR- and Pyrin domain-containing protein 3 (NLRP3) inflammasome activation, autophagy reduces the secretion of pro-inflammatory cytokines (*e.g*., IL-1β, IL-18), thereby mitigating chronic inflammation. Conversely, defective autophagy exacerbates inflammatory responses, further aggravating insulin resistance in women with PCOS (39).

### 3.3 Ferroptosis

Ferroptosis, an iron-dependent form of regulated cell death driven by lipid peroxidation, may play a key role in the pathogenesis of PCOS. In PCOS patients, ovarian granulosa cells frequently exhibit elevated oxidative stress markers (*e.g*., malondialdehyde, MDA) alongside reduced activity of antioxidant enzymes (*e.g*., superoxide dismutase, SOD, and glutathione peroxidase 4, GPX4). This imbalance renders granulosa cells vulnerable to ferroptosis, which can impair estrogen synthesis and disrupt follicular development (40). Hyperinsulinemia, a common feature of PCOS, exacerbates this process by upregulating transferrin receptor 1 (TFR1), thereby enhancing cellular iron uptake. Iron overload, in turn, worsens mitochondrial dysfunction and oxidative stress, creating a vicious cycle that further increases PCOS susceptibility (41). Additionally, the inflammatory microenvironment within the ovary—characterized by elevated levels of cytokines such as TNF-α and IL-6—can activate the NF-κB pathway, suppressing System Xc^-^ expression and promoting lipid peroxidation, ultimately triggering ferroptosis (42).

Notably, androgen excess, a hallmark of PCOS, may also contribute to ferroptosis. Animal studies demonstrate that dihydrotestosterone (DHT) treatment upregulates ferroptosis-related markers (*e.g*., ACSL4) in ovarian cells, suggesting that androgens may exacerbate ferroptosis by modulating lipid metabolism genes (43). The resulting accumulation of (ROS further disrupts ovarian function, exacerbates insulin resistance, and heightens PCOS susceptibility (44–47). Collectively, these findings indicate that ferroptosis contributes to ovarian dysfunction and systemic metabolic disturbances in PCOS through mechanisms involving oxidative damage, metabolic dysregulation, and inflammatory responses.

### 3.4 Epigenetic modifications

Growing evidence has elucidated the critical role of epigenetic modifications in the pathogenesis and progression of PCOS. These heritable molecular alterations regulate gene expression patterns and influence key pathophysiological processes, including ovarian dysfunction, hormonal dysregulation, and insulin signaling impairment, without modifying the underlying DNA sequence (48, 49).

Regarding DNA methylation, studies have demonstrated hypermethylation of the insulin receptor (INSR) promoter region in both ovarian and adipose tissues of PCOS patients, resulting in downregulated insulin receptor expression and aggravated insulin resistance (50). Furthermore, elevated methylation levels of peroxisome proliferator-activated receptor gamma (PPARG) suppress its transcriptional activity, impairing adipocyte differentiation and glucose homeostasis, which contributes to the development of obesity and metabolic syndrome in PCOS patients (51). In ovarian granulosa cells, hypermethylation of the steroidogenic enzyme cytochrome P450 family 19 subfamily A member 1 (CYP19A1) promoter reduces estrogen biosynthesis while relatively increasing androgen production, thereby exacerbating hyperandrogenemia (52). Notably, both hyperandrogenemia and chronic low-grade inflammation can modulate DNA methyltransferase (DNMT) activity, leading to aberrant methylation of the AMH promoter in granulosa cells and subsequent impairment of folliculogenesis in PCOS (50).

Concerning histone modifications, granulosa cells from PCOS patients exhibit enhanced histone deacetylase (HDAC) activity, which decreases acetylation levels of pro-inflammatory cytokines (*e.g*., TNF-α, IL-6) and suppresses their transcriptional activation. This epigenetic dysregulation perpetuates local inflammatory responses and accelerates follicular atresia (53).

Non-coding RNAs also contribute to PCOS pathogenesis. For instance, dysregulated miR-93-3p targets the PI3K/AKT signaling pathway, disrupting granulosa cell proliferation and apoptosis and ultimately arresting follicular development (54). Additionally, androgen excess upregulates miR-223, which suppresses glucose transporter type 4 (GLUT4) expression and worsens insulin resistance (55).

Above all, Epigenetic dysregulation in PCOS—including DNA methylation, histone modifications, and non-coding RNA alterations—disrupts ovarian function, hormone balance, and insulin signaling, contributing to hyperandrogenism, metabolic dysfunction, and impaired folliculogenesis.

### 3.5 Protein phosphorylation

Protein phosphorylation, a critical post-translational modification, plays a pivotal role in the pathogenesis of PCOS by modulating signal transduction, enzyme activity, and cellular functions. In PCOS patients, hyperphosphorylation of serine residues (*e.g*., Ser307, Ser636) on IRS-1/2 suppresses their tyrosine phosphorylation, thereby impairing the downstream PI3K/AKT signaling pathway. This disruption inhibits GLUT4 membrane translocation, exacerbating insulin resistance (56). Additionally, inflammatory cytokines such as TNF-α and IL-6 activate JNK and IKKβ kinases, further promoting IRS-1 serine phosphorylation and creating a vicious cycle that perpetuates insulin signaling dysfunction (57).

Phosphorylation-mediated regulation of steroidogenic enzymes also contributes to PCOS progression. 17α-hydroxylase (CYP17A1), the rate-limiting enzyme in androgen biosynthesis, is modulated by PKA and MAPK-dependent phosphorylation. In ovarian stromal cells of PCOS patients, LH enhances CYP17A1 phosphorylation via the cAMP/PKA pathway, upregulating 17α-hydroxylase and 17,20-lyase activities and leading to excessive production of androstenedione and testosterone (58). Conversely, FSH normally promotes granulosa cell proliferation and estrogen synthesis by inducing tyrosine phosphorylation of the FSH receptor (FSHR). However, in PCOS, reduced FSHR phosphorylation contributes to impaired follicular development and estrogen deficiency (59).

Moreover, excessive LHCGR activation may trigger premature luteinization of granulosa cells through ERK1/2-mediated phosphorylation, disrupting dominant follicle selection. In PCOS granulosa cells, elevated phosphorylation of the pro-apoptotic protein BAX, coupled with suppressed phosphorylation of the anti-apoptotic protein BCL-2, enhances mitochondrial pathway apoptosis, accelerating follicular atresia (60). Collectively, these findings highlight how dynamic protein phosphorylation governs insulin signaling, steroidogenesis, folliculogenesis, and inflammatory responses, underscoring its central role in PCOS pathophysiology.

### 3.6 Chronic low-grade inflammation

Chronic low-grade inflammation have been identified as a central component in the pathogenesis of PCOS, rather than mere accompanying phenomena. Chronic low-grade inflammation is a key contributor to PCOS pathogenesis. Chronic inflammation leads to dysfunction of the endometrium by influencing the proliferation and apoptosis of endometrial cells, thereby increasing the risks of endometrial hyperplasia, PCOS, and cancer (61). The local inflammatory microenvironment in the ovary, characterized by elevated levels of pro-inflammatory cytokines such as IL-18 and IL-1β, impairs the function of follicular granulosa cells and accelerates follicular atresia, ultimately contributing to polycystic-like morphological changes in the ovary (62). Furthermore, oxidative stress and inflammatory mediators, including TNF-α, act synergistically to disrupt mitochondrial function, compromising oocyte maturation in PCOS (63). Additionally, Inflammatory factors, including TNF-α and IL-6, inhibit tyrosine phosphorylation of insulin receptor substrates (IRS), thereby disrupting the insulin signaling pathway and diminishing insulin sensitivity. Inflammatory mediators also enhance androgen synthesis by upregulating steroidogenic enzymes such as steroidogenic acute regulatory protein (StAR) and CYP17A1 (28, 64). Above all, chronic inflammation leads to the occurrence and development of PCOS through multiple pathways such as affecting ovarian function, androgen synthesis, and insulin resistance. Against a background of genetic predisposition, environmental factors such as diet and stress trigger and sustain a chronic low-grade inflammatory state characterized by an imbalance between innate and adaptive immunity. This inflammatory state interacts reciprocally with hyperandrogenism, insulin resistance, and metabolic dysregulation, forming a self-reinforcing vicious cycle. Ultimately, this network drives ovarian dysfunction and a range of clinical manifestations.

### 3.7 Gut microbiota

The gut microbiota, often referred to as the human body’s “second genome” (65), plays a pivotal role in the pathogenesis of polycystic ovary syndrome (PCOS) through multiple pathways, including metabolite interactions, immune modulation, and endocrine signaling (66, 67). One key mechanism involves the gut microbiota’s regulation of bile acid metabolism via bile acid hydrolases and 7α-dehydrogenase, which convert primary bile acids into secondary bile acids. In PCOS patients, an overabundance of Bacteroides vulgatus disrupts the farnesoid X receptor (FXR) signaling pathway, leading to bile acid pool dysregulation and exacerbating insulin resistance and ovarian dysfunction (68).

Additionally, PCOS is associated with an increased abundance of Gram-negative bacteria (*e.g*., Enterobacteriaceae), which produce lipopolysaccharide (LPS). Elevated LPS levels compromise intestinal barrier integrity by downregulating tight junction proteins (*e.g*., ZO-1, occludin), resulting in intestinal permeability (“leaky gut”). Once translocated into systemic circulation, LPS activates toll-like receptor 4 (TLR4), triggering NF-κB-mediated release of proinflammatory cytokines (*e.g*., TNF-α, IL-6) and further aggravating insulin resistance and ovarian inflammation (69).

Sex hormone metabolism is also modulated by the gut microbiota. Through β-glucuronidase activity, gut microbes deconjugate bound androgens, increasing circulating free testosterone levels (70). In PCOS patients, the enrichment of Bacteroides and Prevotella species may exacerbate hyperandrogenemia via this mechanism (71). Furthermore, microbial β-glucuronidase and aromatase activities influence estrogen reabsorption and synthesis, leading to estrogen level fluctuations and hypothalamic-pituitary-ovarian (HPO) axis dysregulation (72).

Clinically, PCOS patients exhibit reduced gut microbial α-diversity, an elevated Bacteroidetes-to-Firmicutes ratio, and decreased abundance of beneficial bacteria such as Akkermansia muciniphila. These alterations correlate significantly with BMI, testosterone levels, and homeostatic model assessment of insulin resistance (HOMA-IR) (73). The gut microbiota also impacts neuroendocrine regulation: microbially derived neurotransmitters, including γ-aminobutyric acid (GABA) and serotonin (5-HT), can modulate hypothalamic GnRH pulsatility via vagal signaling. Notably, in PCOS patients, increased levels of GABA-producing genera (Bacteroides, Prevotella, and Escherichia) are positively associated with elevated serum LH/FSH ratios (74). Collectively, the gut microbiota acts as a critical nexus linking PCOS-related metabolic disturbances to reproductive dysfunction through its metabolites, immune interactions, and neuroendocrine networks.

## 4 The therapeutic targets and drugs of PCOS

Given the multifactorial pathogenesis of PCOS, therapeutic strategies rarely involve a single intervention. Current clinical practice emphasizes personalized treatment plans tailored to each patient’s predominant clinical manifestations. Evidence suggests that combination therapy integrating lifestyle modifications with pharmacological interventions yields superior metabolic outcomes compared to monotherapy, demonstrating significant improvements across multiple metabolic parameters and associated comorbidities.

### 4.1 Advanced glycation end products

AGEs play multifaceted roles in the pathogenesis of PCOS (75–77). AGEs contribute to hyperandrogenism in PCOS by modulating the activity of key steroidogenic enzymes, including CYP11A, CYP17A1, and 3β-HSD (78). Additionally, AGEs disrupt intracellular insulin signaling and glucose transport in human granulosa cells, impairing ovarian function and follicular development (79). Notably, AGEs deposition is a hallmark feature across all PCOS phenotypes. The binding of AGEs to their membrane receptors triggers downstream signaling pathways, promoting oxidative stress, chronic inflammation, hyperandrogenemia, insulin resistance, and anovulatory dysfunction (76, 80).

Emerging therapeutic strategies focus on reducing AGEs accumulation to alleviate PCOS symptoms. Potential interventions include pharmacological agents (*e.g*., aminoguanidine, metformin, phenylthiazolidine, and pyridoxamine), dietary modifications (*e.g*., low-AGE diets), and regular physical exercise (75, 81, 82). Natural compounds, such as green tea polyphenols, exhibit potent anti-AGE activity, surpassing even aminoguanidine in efficacy (83). Therefore, developing anti-AGE molecules, adopting AGE-restricted diets, and incorporating exercise regimens may offer innovative approaches to mitigate AGEs-induced fertility impairments in PCOS patients.

### 4.2 SHBG

SHBG is a plasma glycoprotein primarily synthesized in the liver that binds to androgens and estrogens, regulating their bioavailability to target tissues. Circulating SHBG levels are inversely correlated with markers of non-alcoholic fatty liver disease (NAFLD) and insulin resistance (84). Reduced SHBG levels increase the bioavailability of androgens, contributing to ovarian dysfunction, anovulation, and the phenotypic manifestations of PCOS (85). Additionally, insulin resistance induces hyperinsulinemia, which further suppresses SHBG production, exacerbating reproductive dysfunction. Notably, insulin-sensitizing therapies (*e.g*., metformin) have been shown to elevate SHBG levels in women with PCOS, establishing SHBG as a reliable biomarker for insulin resistance (86, 87). Obesity, particularly during puberty, plays a critical role in PCOS development. Childhood obesity is an early indicator of insulin resistance and a predisposing factor for PCOS, partly due to obesity-mediated reductions in SHBG synthesis, which enhance testosterone bioavailability (88).

Combined oral contraceptives (COCs) are first-line treatments for PCOS, effectively lowering androgen levels and restoring menstrual regularity. For instance, COCs containing 30 μg ethinyl estradiol and 3 mg norgestrel have been shown to increase plasma SHBG from 37.31 nmol/L to 179.01 nmol/L. However, COC use is also associated with impaired fasting glucose, insulin resistance, and an elevated risk of thromboembolic events (85). Given its role in hormonal and metabolic regulation, SHBG serves as a valuable biomarker for early PCOS detection and intervention.

### 4.3 miRNA

Growing evidence highlights the therapeutic potential of miRNAs in PCOS (47, 89–92). Studies have identified differential expression of specific miRNAs in women with PCOS compared to healthy controls, suggesting a key regulatory role in the pathogenesis and progression of the disorder. Aberrant miRNA expression has been observed in multiple tissues and biological fluids, including follicular cells, adipose tissue, follicular fluid, cumulus cells, granulosa cells, serum, and peripheral blood leukocytes of PCOS patients (93).

In PCOS, miRNAs modulate critical processes such as steroid hormone synthesis [84], follicular development and maturation (94), fatogenesis (95), and insulin signaling pathways (96). These dysregulations contribute to inflammation, impaired ovarian insulin sensitivity, hyperinsulinemia, and compromised oocyte quality. Notably, miRNAs influence oocyte development and follicular growth by regulating ovarian steroidogenesis, cell proliferation, and apoptosis, positioning them as potential biomarkers for assessing ovulatory dysfunction in PCOS (97).

Furthermore, miRNAs play a pivotal role in cholesterol homeostasis and lipid metabolism. Key miRNAs associated with low-density lipoprotein cholesterol (LDL-C) metabolism, adipogenesis, and BMI—including miR-21, miR-27b, miR-103, miR-155, miR-24, miR-29, and miR-502-3p—are dysregulated in PCOS (98, 99). Given the strong link between miRNA dysfunction, obesity, and dyslipidemia, targeting miRNAs presents a promising therapeutic strategy for alleviating metabolic complications in PCOS (100).

Potential miRNA-based interventions include restoring or suppressing miRNA activity using synthetic mimics or inhibitors (anti-miRs) (101, 102). Although no miRNA-targeting drugs are currently approved for PCOS, ongoing research into miRNA-related biomarkers offers novel avenues for developing precision therapies.

## 5 Conclusion

PCOS arises from the complex interplay of multiple factors, including genetic predisposition, metabolic disturbances, endocrine abnormalities, immune imbalance, and environmental influences. Clinically, PCOS patients typically present with ovarian dysfunction and dysregulated glucose and lipid metabolism. Emerging evidence suggests that the underlying pathophysiology involves oxidative stress, impaired autophagy, ferroptosis, protein post-translational modifications, chronic low-grade inflammation, and gut microbiota dysbiosis affecting ovarian, hepatic, and pancreatic tissues. Given the multifactorial nature of PCOS, therapeutic approaches require personalized strategies rather than single-modality interventions. Current management combines lifestyle modifications (anti-inflammatory diet, exercise) with pharmacological therapies targeting specific disease manifestations to address the diverse clinical presentations of PCOS. This comprehensive review elucidates the pathogenic mechanisms and identifies potential preventive targets for PCOS, thereby establishing a scientific foundation for developing effective treatment strategies. Future research directions may include the development of targeted drugs based on recently identified molecular targets such as AGEs, SHBG, and miRNAs, which show promising therapeutic potential.
